# Context-driven communication during deep-sea foraging in a social toothed whale

**DOI:** 10.1098/rsos.240558

**Published:** 2024-07-31

**Authors:** Sanne Hessing, Nolwenn Risser, Loanne Pichot, Machiel G. Oudejans, Marie Guilpin, Luís M. D. Barcelos, Charlotte Curé, Fleur Visser

**Affiliations:** ^1^ Kelp Marine Research, Hoorn 1624 CJ, The Netherlands; ^2^ Department of Coastal Systems, Royal Netherlands Institute for Sea Research, PO Box 59, Den Burg 1790 AB, The Netherlands; ^3^ Cerema-University Gustave Eiffel, UMRAE, Acoustics Group of the Laboratory of Strasbourg, Strasbourg F-67210, France; ^4^ Département de Biologie, ENS École Normale Supérieure de Lyon, Lyon Cedex 07 69342, France; ^5^ Azorean Biodiversity Group & Center for Ecology, Evolution and Environmental Changes & CHANGE—Global Change and Sustainability Institute, University of the Azores, 9700-042 Angra do Heroísmo, Terceira, Azores, Portugal

**Keywords:** animal communication, biologging, burst-pulse, deep-diving odontocete, foraging behaviour, *Grampus griseus*

## Abstract

Social deep-diving odontocetes face the challenge of balancing near-surface proximity to oxygen and group members with foraging in the deep sea. Individuals rely on conspecifics for critical life functions, such as predator defence, but disperse during foraging to feed individually. To understand the role of social acoustic mediation during foraging in deep-diving toothed whales, we investigated the context of social burst-pulse call production in Risso’s dolphin (*Grampus griseus*) using biologgers. Dolphins produced context-specific burst pulses predominantly during daytime foraging, preceding or following foraging dives and in the early descent of daytime deep dives. Individuals applied differential short and long burst-pulse calls intended for either near-surface receivers (horizontal transmission) or deep-foraging receivers (vertical transmission). Our results show that deep-diving toothed whales are reliant on acoustic communication during certain foraging contexts, to relay information including foraging conditions or an individual’s location. Moreover, they accentuate the importance of maintaining acoustic contact with conspecifics, specifically when dispersed during deeper foraging. It also signifies that our oceanic top predators may be specifically vulnerable to the current strong increase in anthropogenic noise. Potential masking of the signals from group members communicating at a distance could undermine their social cohesion, and hence their capacity to maintain vital life functions.

## 1. Introduction

Cetaceans rely on acoustics for vital functions such as navigation, communication, foraging and predator avoidance [1,2]. Being highly dependent on sound, cetaceans are vulnerable to anthropogenic underwater noise. Behavioural disturbances (e.g. disrupted foraging), acoustical disruption (e.g. adapted call use or masking) or physical damage (e.g. temporary or permanent hearing loss) could lead to reduced fitness and survival [3]. However, the acoustic communication systems of cetaceans, e.g. required for relocation of conspecifics following periods of dispersal, are often poorly understood, and we have yet to understand their context of production and specific function.

Social deep-diving toothed whales form highly cohesive social groups at the surface [4,5]. During foraging, however, group members spread out to feed individually at depth [6–8]. These deep divers must balance the benefits of foraging in remote deep-sea waters for capturing energetic prey with the critical need for oxygen access and proximity to their conspecifics. Acoustic communication is expected to be the primary mediator in maintaining group cohesion and enabling relocation at the surface [9]. The importance of staying connected with conspecifics has been demonstrated in Blainville’s beaked whales (*Mesoplodon densirostris*) that produce social calls only at great depth when they separate to forage while remaining in close coordination with their calves [10–12]. Pilot whales (*Globicephala* sp.) increase their call rates during foraging [13] and are capable of producing social calls at great depth [14]. In sperm whales (*Physeter macrocephalus*) [15–17] and Risso’s dolphins (*Grampus griseus*) [18,19], social calls were recorded both at the onset of and during the foraging dive. The specific conditions that provoke the production of these sounds and their functional importance remain unknown.

To better understand the potential role of social acoustic mediation during foraging, we investigated the contextual use and timing of social burst-pulse calls produced within a foraging context in Risso’s dolphins. Risso’s dolphins are highly social deep-diving tooth whales, which in contrast to several other highly social delphinids, show relatively sparse production of social calls (F Visser 2021, personal communication). Therefore, they provide the opportunity to create insight into the contextual usage of burst-pulse calls during foraging. Off the Azores, Risso’s dolphins’ foraging dives trace the depth of the deep scattering layer. Individuals use different foraging strategies. During the day, they perform spin dives to target deep layers, and during night-time, they perform more shallow non-spin dives [20]. In addition to the variation in diving strategies, Risso’s dolphins produce a range of social calls, including whistles, burst pulses and the simultaneous production of a whistle and a burst pulse, termed a whistle burst pulse [18,19,21,22]. Highly directional broadband burst pulses are acoustic signals that have been proposed to serve a communicative function and have been documented to be associated with foraging context [18,19]. While tonal calls are challenged by hydrostatic pressure, click-based sounds, such as the burst pulse, appear to be a suitable communicative signal for deep-diving odontocetes since they require less air volume [23]. We hypothesize that Risso’s dolphins produce burst-pulse calls (i) during or around foraging dives, to inform group members about their location; (ii) during deeper dives, to remain connected with group members while being most dispersed; and (iii) during night-time as the potential use of visual cues is impeded.

## 2. Material and methods

### 2.1. Acoustic dataset

We analysed the occurrence of burst-pulse vocalizations throughout the dive cycle by examining nine existing tag records (DTAG v. 3) [24], holding periods of foraging behaviour, deployed on eight individual Risso’s dolphins (one animal was tagged twice) off Terceira Island, Azores, between 2013 and 2019 (table 1). DTAGS were deployed using a 6–8 m carbon-fibre pole from a small 6 m rigid-hulled inflatable boat [20]. Tags were attached non-invasively with four suction cups and detached after 4.9–16.7 h (table 1). Detached tags floated to the surface and were retrieved using VHF radio tracking [24]. DTAGs recorded acoustics using two built-in hydrophones (sampled at 240 kHz) and diving depth, using a pressure sensor (sampled at 200 Hz) [24], allowing for the identification of foraging dives [25] and matching of the timing of social call production with the dive cycle.

**Table 1.** Tag data summary. Number of foraging dives and burst-pulse (BP) calls during nine tag deployments on Risso’s dolphins (*G. griseus*) off Terceira Island, Azores. Dive categories: SD = spin day, SN = spin night, NSD = non-spin day, NSN = non-spin night. Phases of dive cycle: PRE = 0–16 min preceding the foraging dive, DES = descent phase, from surface till first buzz, FOR = foraging, between first and last buzz, ASC = ascent, from last buzz till resurfacing, POST = 0–16 min following the foraging dive.

whale ID	tag ID	tag date	tag on time (h)		duration (h)	no. of foraging dives (complete)	no. of dives per category SD/SN/NSD/NSN	no. BP dive cycle PRE/DES/FOR/ASC/POST	
1	Gg13_238 a	26 Aug 2013	16.12		5.7	6 (5)	2/0/1/3	6/2/0/4/15	
1	Gg17_203 a	22 July 2017	12.33		9.4	20 (20)	17/3/0/0	50/23/0/5/21	
2	Gg15_229 a	17 Aug 2015	09.41		16.7	41 (41)	7/3/0/31	51/7/0/4/38	
3	Gg15_229 c	17 Aug 2015	13.53		11.0	19 (19)	0/0/0/19	0/0/0/3/3	
4	Gg16_169 a	17 June 2016	14.12		4.9	9 (9)	8/0/1/0	14/9/0/8/37	
5	Gg16_171 a	19 June 2016	08.51		11.8	4 (4)	4/0/0/0	3/0/0/1/14	
6	Gg17_200 a	19 July 2017	11.04		15.9	27 (26)	4/22/0/1	15/7/0/0/4	
7	Gg18_214 a	02 Aug 2018	15.37		10.2	22 (22)	0/6/0/16	16/1/0/1/7	
8	Gg19_197 a	26 July 2019	09.40		9.5	1 (0)	1/0/0/0	25/6/tag off	
			Total		95.1 h	149 (146)	43/34/2/70	180/55/0/26/139	

### 2.2. Acoustic and depth data processing

The first 15 min of each DTAG recording were excluded from the analysis to avoid potential tagging effects on the behaviour of the tagged animal [19,25]. Pressure sensor data were calibrated and converted to obtain dive depth. Incomplete dives (*n* = 3), lacking the full ascent phase owing to a tag-off event, and the associated burst-pulse calls (*n* = 31) were only included in the analysis of the descent phase. Tags recorded both vocalizations of the tagged Risso’s dolphin as well as from animals nearby. Sounds produced by the tagged animal propagate within the body and create an artificial low-energy component [26,27]. Together with the angle of arrival of the calls on the two hydrophones [24], this enables the distinction between calls produced by the tagged animal from those produced by nearby conspecifics [26,28]. Vocalizations, including foraging echolocation clicks (search clicks), foraging buzzes and social calls, were identified by their acoustic characteristics [19]. Echolocation clicks are broadband signals of short duration (40 µs) with a peak frequency of 50 kHz [29]. Buzz and burst-pulse sounds consist of a series of high-repetition clicks, where a buzz indicates a prey capture attempt, while burst pulses are theorized to serve a communicative function. Buzzes follow regular echolocation click trains [19]. Other social call types produced by Risso’s dolphins include the whistle (frequency-modulated, narrowband tonal sound) and a combination of a burst pulse with a simultaneous whistle, termed whistle burst pulse [22]. Given the known prevalence of the burst pulse as the main call produced in the foraging context, we focused on the burst pulse. The start and end of all vocalizations recorded on the tag were marked manually on the spectrogram (Hamming window, 1024 fast Fourier transform, 512 (50%) overlap, 90 dB dynamic range), and classified by (i) call type and (ii) produced by the tagged animal or other nearby conspecifics. Calls marked as burst pulse and produced by the focal animal were extracted and overlaid on the diving profile. To explore whether burst-pulse characteristics are associated with contextual use, the occurrence and duration of the call were examined in relation to timing within the dive cycle (i.e. the different phases of the dive). Call length was obtained by using an automated *findclicks* function, within the DTAG toolbox, in which the automated detection was manually checked and adjusted, to make sure all clicks within an isolated call were selected. In addition, the maximum dive depth, start of clicking and burst-pulse depth were determined to investigate the relation of call use with foraging diving depth. Acoustic and depth sensor data from the DTAGs were processed in Matlab R2013a (MathWorks, Natick, MA, USA) using the DTAG toolbox (soundtags.wp.st-andrews.ac.uk).

### 2.3. Foraging context and dive type specification

**Figure 1.**
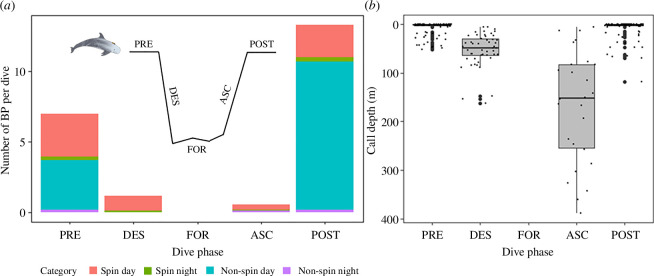
Burst-pulse (BP) production as a function of phase in the dive cycle. (*a*) Mean number of BP recorded per dive over the different dive phases, for each of the four foraging dive categories (colours). (*b*) Depth of burst-pulse production by dive phases. Boxplots present the median (solid horizontal line), the interquartile range (boxes), values 1.5 × the interquartile ranges (whiskers), outliers (thick dots) and raw data points (thin dots). PRE = 0–16 min preceding the foraging dive; DES = descent phase, from surface till first buzz; FOR = foraging, between first and last buzz; ASC = ascent, from last buzz till resurfacing; POST = 0–16 min following the foraging dive.

Foraging dives were defined as dives deeper than 20 m [25], containing at least one buzz vocalization, i.e. indicating a prey capture attempt [19,20]. Non-foraging dives were defined as all dives deeper than 20 m and without a buzz vocalization. All foraging dives were then classified as spin dives or non-spin dives, following Visser *et al*. [20]. Spin dives are deep-foraging dives (mean (s.d.) = 428 (131) m; range 132–623 m), employed mostly during the daytime and characterized by a strong acceleration and right-turning rotation (spin) at the dive onset. Non-spin dives are shallower foraging dives (mean (s.d.) = 185 (88) m; range 37–599 m) and start with an arch-out (slow-rolling dive onset). Foraging dives were classified as ‘day’ or ‘night’ by the timing of the dive onset relative to local sunset in the Azores (Portugal). This resulted in the following four foraging dive categories: spin day, spin night, non-spin day and non-spin night. A foraging bout was defined as a series of foraging dives with a maximum inter-dive interval of twice the mean foraging dive duration (16 min; mean (s.d.) = 8 (2) min, *n* = 146). The foraging period was defined as starting 16 min before the first foraging dive and ending 16 min after the last foraging dive of a foraging bout, to include calls potentially facilitating foraging onset and relocation after foraging, at the surface. To investigate calling behaviour as a function of foraging behaviour, all burst-pulse calls within foraging periods were analysed for their occurrence in relation to the phase of the dive cycle, dive type and time of day. Calls were assigned to dive cycle phases according to their production timing, and defined as follows: (i) calls emitted during the 0–16 min preceding the foraging dive (PRE); (ii) during the descent (DES), the period between the surface and the first emitted buzz; (iii) during foraging (FOR), the period between the first and the last buzz; (iv) during the ascent (ASC), the period between the last buzz and resurfacing; or (v) during the 0–16 min following the foraging dive (POST; figure 1*a*). A call occurring between two foraging dives was classified as PRE or POST based on the smallest time difference to the nearest dive.

### 2.4. Statistical analysis

To investigate whether the occurrence of burst-pulse calls differed as a function of timing within the dive cycle, dive type and time of day, we used generalized estimating equations (GEEs) [30], in R v. 4.0.3 [31]. GEEs were fitted using the *geepack* package [32]. To account for the repeated measures design, whale ID was used as a clustering factor, which allowed residuals to correlate within an individual but were assumed to be independent between individuals. The models were fitted with a Gaussian family identity link and an autoregressive correlation structure (AR1) was used to correct for temporal autocorrelation [33]. This implies that the expected correlation between the occurrence of burst-pulse calls within each cluster (whale ID) decreased when the burst-pulse calls got further apart in time. To test whether Risso’s dolphins’ use of burst pulses varied across specific phases of foraging dives (hypothesis 1), burst-pulse production was fitted as a response variable and dive phase (PRE, DES, ASC and POST) as the predictor variable (GEE1). In addition, the context of call duration was investigated by which burst-pulse length was fitted as the response variable and the timing within the dive cycle (at the surface or during the dive) as the predictor variable (GEE2).

To test whether Risso’s dolphins’ burst-pulse use varied across dive types and time of day (hypotheses 2 and 3), burst-pulse production was fitted as a response variable, using dive type (spin and non-spin), time of day (day and night) and their interaction as predictor variables, for all burst pulses (GEE3a), and separately for the burst pulses produced during different phases of the dive (GEE3b–e). The effect of the foraging context was further studied by examining whether maximum diving depth (GEE4) and depth of start of clicking (GEE5) varied as a function of the dive category. A backward model selection was conducted, in which individual predictors with the largest *p*-value in sequential Wald tests using ANOVA (*geepack* package) were removed. The models were refitted until all predictors retained were significant (*p* < 0.05).

## 3. Results

The eight tagged individuals performed 149 foraging dives (95.1 h of tag data; table 1), of which 146 complete dives were included in the analysis. Individuals foraged during both day- and night-time (*n* = 45 versus 104 of dives day and night, respectively), with foraging dive depths ranging from 37 to 623 m (mean (s.d.) = 310 (165) m). During daytime, Risso’s dolphins predominantly performed spin dives (96%, *n* = 43), while during night-time dives, individuals mainly produced non-spin dives (67%, *n* = 70). A total of 400 burst pulses were identified within the foraging context, across eight individuals (table 1). Burst pulses (67% of all social calls, *n* = 400) were found to be the dominant used social call within the foraging context. Other recorded social calls (whistle burst pulse: 25%, *n* = 150; whistles: 8%, *n* = 45) had lower call rates.

### 3.1. Burst-pulse use during and around foraging dives

Burst pulses were emitted during all dive phases except for the foraging phase of the dive (figure 1*a*). Burst-pulse production varied as a function of the dive phase (GEE1, *p* < 0.001; table 2 and electronic supplementary material, table S1) and most calls were emitted around surfacing events, preceding (PRE) and following (POST) foraging dives (figure 1*a*). Post-dive burst-pulse calls were mostly produced within 1 min after resurfacing (*n* = 105/139, 76%). Burst pulses produced at the surface, either at the dive onset or when resurfacing, were found to be significantly shorter than burst-pulse calls produced during the descent and ascent phase (mean ± s.d. = 0.13 ± 0.11 versus 0.34 ± 0.08 s, respectively; GEE2, *p* < 0.001; table 2 and electronic supplementary material, table S2). Burst-pulse production was typically shallow, occurring near the surface pre- or post-diving (mean depth ± s.d. = 6.3 ± 14 m) or early in the descent (mean ± s.d. = 53 ± 35 m; figure 1*b* ). During ascent call, depth was more dispersed and calls were emitted just after the last buzz or near-surface, with a mean depth of 168 m and strong variation in depth (s.d. = 117 m) (figure 1*b*). Burst pulses were not specifically associated with the first or last dive in a foraging bout.

**Table 2.** Results of the ANOVA (sequential Wald tests) showing the significance of the GEE models. Parameter estimates and standard errors (s.e.) of the different models can be found in the electronic supplementary material, tables S1−10. Dive phases: PRE = 0–16 min preceding the foraging dive/DES = descent phase, from surface till first buzz/FOR = foraging, between first and last buzz/ASC = ascent, from last buzz till resurfacing/POST = 0–16 min following the foraging dive. Significance codes: ‘***’ 0.001, ‘**’ 0.01, ‘*’ 0.05.

model	response variable	factor	*χ* ^2^	*p*‐value
GEE1	burst-pulse production	dive phase	41.2	2.4 × 10^−8^***
GEE2	burst-pulse duration	timing within the dive cycle	187	< 2 × 10^16^***
GEE3a	burst-pulse production	time of day	17.2	3.4 × 10^−5^***
GEE3b	burst-pulse production during dive phase: PRE	time of day	15	0.00011***
GEE3c	burst-pulse production during dive phase: DES	dive type	5.14	0.023*
		time of day	17.22	3.3 × 10^−5^***
		dive type : time of day	24.95	5.9 × 10^−7^***
GEE3d	burst-pulse production during dive phase: ASC	dive type	2.23	0.1350
		time of day	7.32	0.0068**
		dive type : time of day	32.4	1.2 × 10^−8^***
GEE3e	burst-pulse production during dive phase: POST	dive type	1.50	0.22116
		time of day	8.47	0.00361**
		dive type : time of day	13.73	0.00021***
GEE4	maximum diving depth	dive category	251	< 2 × 10^−16^***
GEE5	start of clicking depth	dive category	62.5	1.8 × 10^−13^***

### 3.2. Burst-pulse use during deep and shallow foraging

**Figure 2.**
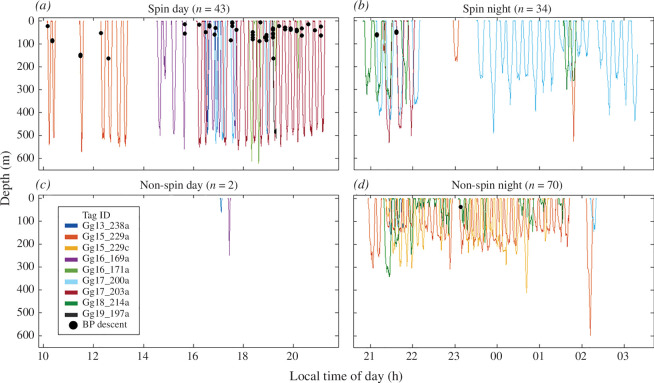
Occurrence of burst pulses (BP; black circle) produced during the descent phase of a foraging dive. The panels show all individuals’ foraging dives per dive category, (*a*) spin day, (*b*) spin night, (*c*) non-spin day and (*d*) non-spin night. Each tag deployment (tag ID) is represented by a colour code.

Dive type was found to be a significant predictor for burst-pulse calls produced during the descent phase, ascent phase and post-diving (GEE3c–e, *p* < 0.05; table 2 and electronic supplementary material, tables S5−7). During spin dives, burst-pulse production was higher during the descent and ascent phases (GEE3c–d, *p* < 0.05; table 2 and electronic supplementary material, tables S5−6), while calls produced following foraging dives (POST) showed higher production rates during non-spin dives (GEE3e, *p* < 0.001; table 2 and electronic supplementary material, table S7). Overall, burst-pulse call rates during spin dives were five times higher (*n* = 309/77 dives) than during non-spin dives (*n* = 60/72 dives). More specifically, Risso’s dolphins showed specific use of burst pulses during descent in the daytime spin dives (GEE3c, *p* < 0.001; table 2 and electronic supplementary material, table S5), and this behaviour was commonly recorded, (67%, 29/43 spin day dives), across six of the seven individuals performing this dive type (figure 2). The seventh individual (Gg16_171 a) only made a limited number of spin dives (*n* = 4) of which two were relatively shallow.

### 3.3. Burst-pulse use during day- and night-time

**Figure 3.**
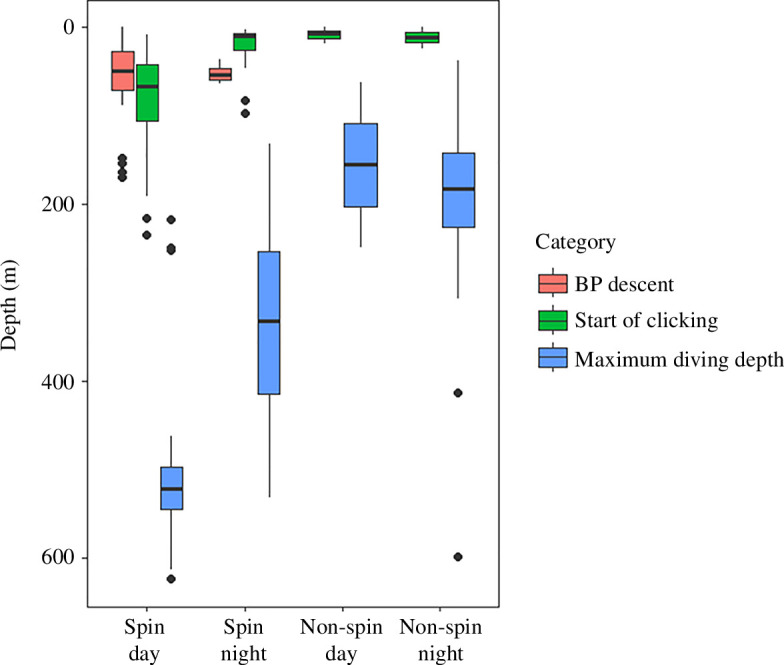
Burst-pulse (BP) production as a function of dive depth and echolocation behaviour. Comparing the depth of BP production during the descent phase (red), depth of start of clicking (green) and maximum diving depth (blue) per dive category. Boxplots present the median (solid horizontal line), the interquartile range (boxes), 1.5 interquartile ranges (whiskers) and outliers (black circles).

Burst-pulse production exhibited pronounced diel variation. Time of day had a significant effect on burst-pulse production (GEE3a, *p* < 0.001; table 2 and electronic supplementary material, table S3) in which the majority of the burst pulses were produced during daytime (85%, *n* = 312 versus 15%, *n* = 57, day and night, respectively; figure 1*a*). Burst-pulse call rates produced during foraging dives were 13 times higher during daytime than during night-time (6.9 versus 0.55 burst pulses per foraging dive, respectively). While clear contextual use of burst-pulse production was recorded during the descent phase of spin day dives, this behaviour remained absent during spin night dives (figure 2). Spin foraging dives showed deeper maximum diving depths over day versus night (mean (s.d.) = 506 (81) versus 329 (114) m; GEE4, *p* < 0.01; table 2, electronic supplementary material, table S8 and figure 3) and started their echolocation clicking later after the dive onset during the day (mean (s.d.) = 81 (56) versus 21 (21) m; GEE5, *p* < 0.01; table 2, electronic supplementary material, table S9 and figure 3). Burst-pulse production during the descent phase of a dive most often occurred before starting echolocation clicking (*n* = 44/55, 80%).

## 4. Discussion

The deep-diving social odontocete, Risso’s dolphin, employed differential communication modes during dispersed foraging, one probably geared for horizontal transfer to near-surface individuals and a second mode for vertical transfer to deeper-diving individuals. Short burst pulses were used at shallow depths during or after surfacing from foraging dives, by the animal swimming at or very near the surface. Long burst pulses were used during the descent or ascent of deep-foraging dives when the individual was angled steeply down or up [20]. Surprisingly, while diving deep, most burst-pulse calls occurred near the surface or within the first 100 m of the dive descent, and predominantly during daytime, when foraging dives were deepest. Burst-pulse use and production rates, as well as call length, were specific to the foraging dive phase, dive type and time of day. These results demonstrate that social toothed whales are likely to use acoustic communication to maintain contact and/or share information with conspecifics to facilitate efficient foraging. Anthropogenic noise has the potential to mask communication between group members and could therefore undermine their social cohesion, and hence their capacity to maintain vital life functions like foraging and predator defence.

### 4.1. Shallow communication during deep foraging

Risso’s dolphins consistently produced burst-pulse communication calls within the foraging context. Calls were predominantly produced during the day, preceding or following foraging dives, or in the early descent or late ascent of foraging dives, respectively, preceding or following the echolocation search phase. They did not call during the prey search and capture phases, probably because they are then already audible by their echolocation search clicks, and producing burst-pulse calls may reduce their foraging efficiency. Although capable of producing tonal sounds such as whistles, Risso’s dolphins favoured the use of highly directional burst pulses within a foraging context, in agreement with previous findings by Neves [18]. Call rates were high near surfacing events at the onset and offset of foraging dives, supporting our hypothesis that Risso’s dolphins communicate near-surface at the start and end of foraging dives. As not all dives contained associated burst-pulse calls and calling was not recorded during the night, Risso’s dolphins probably can also relocate their group members through eavesdropping on each other’s foraging echolocation signals [34] or other potential sensory cues such as chemical signals [35].

### 4.2. Horizontal communication with near-surface group members

Pre- and post-diving burst pulses were significantly shorter (mean duration (s.d.) = 0.13 (0.11) s) than those used during foraging dive descent or ascent (mean (s.d.) = 0.34 (0.08) s), signifying alternative information transfer and communication needs between animals engaged in a foraging dive and those present near the surface. This shorter type of burst-pulse call may serve as a quick and efficient way to acoustically signal position to group members. Transmission of broadband burst-pulse calls is described as highly directional for the frequency components above 5 kHz and becomes attenuated at greater angles [36–38]. Risso’s dolphin produces broadband clicks with a peak frequency of around 50 kHz and little energy below 20 kHz [29]. Surfacing animals would probably be able to signal their position to listeners as these broadband signals make good carriers of location and direction of movement as directionality alters the signal structure (absence of higher frequency components when the signaller is moving away) [36]. The difference in attenuation will allow receivers to deduce the signallers’ orientation, and subsequently, these cues may be important to relocate and improve cohesion during foraging behaviour. Given the production at or near surfacing events, the vocalizing individual is probably oriented more or less horizontally, suggesting most energy is directed in the horizontal plane, and that the signal is intended for other near-surface individuals.

### 4.3. Deep-foraging dives demand specific communication

Foraging dive depth of Azorean Risso’s dolphins traces the diel vertical migration of the deep scattering layer. They use two different foraging dive types: spin dives with sprints to target deep layers (500–700 m), and more shallow non-spin dives focusing on shallow-residing prey, which has migrated closer to the sea surface at night [20]. These dive types provide two foraging contexts, in which the relative distribution of and distance between group members is likely to differ substantially and demand different communication strategies. The second mode of foraging dive communication occurred during the diving descent, or—less often—the dive ascent, of daytime deep dives. Descent-phase burst pulses were produced early in the descent and at a defined depth (mean (s.d.) = 53 (35) m), predominantly before starting an echolocation-guided prey search. Producing a burst-pulse call, just before starting with echolocation clicking, suggests a communicative function. This specific timing of call use has also been reported in Blainville’s beaked whales (*M. densirostris*) [10,11], in which these calls were suggested to coordinate group dispersion at depth. For Risso’s dolphins, the signal probably serves a different communication function (detailed below) as they demonstrate dispersion already at the surface and remain vocal also in shallow waters.

Burst pulses were previously described for spinner dolphins (*Stenella longirostris*) as a more intimate form of signalling meant for conspecific in close vicinity [37], while studies on odontocetes using narrowband high-frequency clicks theorize that broadband burst pulses may be suitable for long-range detection as the low-frequency component transmits further and can be detected more readily by conspecifics [38–41]. Sound level differences as large as 40 dB were reported to occur in orientation between 0 and 180 degrees relative to the bottlenose dolphins’ front [38]. Shading from cranial bones and nasal air sacs also plays a role, making it difficult for a broadband signal to radiate equally in all directions [42,43]. This indicates that conspecifics at depth are the intended receivers and would receive the high-amplitude, high-frequency component of the signal, while other dispersed group members (at the surface or elsewhere) might only receive the low-frequency, omnidirectional component of the signal [44]. The longer duration of burst-pulse calls during the dive descent indicates a requirement for the call to transfer its information over longer distances (against signal transmission loss) [45], or the need for higher information content.

Surprisingly, Risso’s dolphins only used burst pulse during the descent phase of deep spin dives, but not during non-spin dives or at night when the spin dives were shallower. A comparable pattern was observed for short-finned pilot whales, which produced most social calls in relation to foraging dives deeper than 120 m and, to a lesser extent, associated with shallower foraging dives (less than 73 m) [13]. During shallower dives, burst-pulse calls remained absent, probably because Risso’s dolphins may be less dispersed and consequently will have less need to call for relocating each other. Alternatively, Risso’s dolphins might communicate about the presence or quality of prey with conspecifics at depth [46]. Spin dives, aiming for remote, deep residing, dispersed prey fields, require more energy owing to the costly rotation sprint, and Risso’s dolphins may have higher benefits from sharing prey information and tracking mobile prey once discovered, compared with shallower dives [20].

Risso’s dolphins produce high numbers of burst-pulse signals within the foraging context and probably benefit from increased cohesion for relocation and sharing information regarding prey, during and after separate foraging. The nature of what is being communicated remains to be understood. Odontocetes that use narrowband high-frequency clicks for communication, like harbour porpoises (*Phocoena phocoena*) [39] or Heaviside’s dolphins, (*Cephalorhynchus heavisidii*), can encode specific information such as identification using stereotyped burst pulse or codas [41]. It could be that the burst pulse also holds an identity component.

### 4.4. Reduced burst-pulse call production after dusk

Acoustic communication is believed to have evolved in association with variation in light, as when visibility is reduced (at night or in marine environments), sounds have higher efficiency compared with visual cues [47,48]. We hypothesized that Risso’s dolphins would be more communicative at night. This behaviour has been reported for different species, such as Guiana dolphins (*Sotalia guianensis*) [45], short-beaked common dolphins (*Delphinus delphis*) [49] and the Risso’s dolphin [50], for tonal calls. However, we found the opposite and recorded a strong reduction in the use of burst-pulse production after dusk. The reason behind the variation could be caused by a difference in species, call type, methods (tagging versus hydrophone) or study site, as ecological factors such as the prey scape appear to influence call use. Species targeting light-limited or deprived waters for foraging may use comparable social acoustic strategies during foraging. A comparable diel variation in call production was observed in other deep-diving cetaceans, including sperm whales, in which lower social call rates (codas) were recorded during the night compared with the day. However, this pattern was not directly associated with foraging [17]. In addition, melon-headed whales (*Peponocephala electra*) also showed a strong decline in whistle use at the beginning of the night when they started foraging on vertically migrating mesopelagic prey [51]. This raises the question of how individuals relocate each other and share information in the dark. While day and night hold different light regimes, this is much less prevalent in the Risso foraging grounds, which are always at low/no light conditions. What is profoundly different at night is the shallower diving depth (i.e. vertical dispersion range from the surface and conspecifics) and the prey scape, holding vertically migrating prey that is likely to be more active when feeding in shallower water, than during the day when they respire, defecate and excrete at depth. This shift could release the communication need, possibly because group members can stay closer together (and rely on eavesdropping) without foraging interference.

There could be an increased need to stay in touch during the daytime as they dive to greater depths. Future studies could involve tagging multiple individuals of the same group overnight or analysing the received level of congener echolocation clicks to test whether during nocturnal foraging they show the same spatial distribution as during daytime.

Another argument could be that the background noise properties of daytime versus night-time are more or less favourable for acoustic communication (biological masking and masking by man-made sounds). Risso’s dolphins forage in dense biomass aggregations, called scattering layers, which show diel vertical migrations, moving up around dawn and down again during dusk [20]. This diel vertical migration of biomass can potentially contribute to higher ambient sound levels in the upper layers of the ocean, owing to e.g. snapping shrimp [52].

We show that a social deep-diving odontocete, the Risso’s dolphin, employs differential modes of communication when foraging. During foraging contexts with a high degree of dispersion, foraging individuals communicate both horizontally and vertically. We propose that these signals not only function to maintain group cohesion but also to coordinate synchronized but dispersed foraging on cryptic, remote and ephemeral prey. Night-time conditions with altered, more proximate and possibly denser hunting grounds release the need for foraging communication, and individuals can remain in contact through passive listening to each other’s foraging echolocation signals. These data provide insight into the complex socio-environmentally driven foraging behaviour and social needs of deep-diving toothed whales, signifying that the ability to successfully receive a variety of acoustic cues is critical to maintaining behaviours important for individual fitness and ultimately population health.

## Data Availability

Data supporting this manuscript are accessible in the Dryad Digital Repository [53]. The GEE model results are provided in the supplementary material [54].
